# From the lecture theatre to your digital device: Reflections on the production of educational podcasts within undergraduate psychiatry training

**DOI:** 10.1192/j.eurpsy.2021.2197

**Published:** 2021-08-13

**Authors:** J. Rahman, A. Charalambous, L. Aled, X. Leonard, C. Parsons, G. Cole, G. Sharma, K. Skuse, M. Tran

**Affiliations:** Pyrland Ward, Somerset Foundation Trust, Taunton, United Kingdom

**Keywords:** podcast, training, peer-to-peer, undergraduate

## Abstract

**Introduction:**

The COVID-19 pandemic has highlighted a need for engaging online resources to enrich psychiatry training for undergraduate medical students. Podcasting is a well-established digital communication platform utilised daily in a myriad of capacities, including education. A group of medical students were tasked with creating their own educational podcasts covering specific aspects of psychiatry.

**Objectives:**

Each pair was set a sub-topic of psychiatry and utilised software to produce educational resources. The objective of this project was to reflect upon production as well as explore the efficacy of podcasting as a tool within undergraduate training.

**Methods:**

The medical students conducted research and contacted experts within the field to contribute to their podcasts. The majority of the students then conducted reviews of the literature surrounding podcasting within medical education, which informed the production of their own podcasts. From this, it was discussed how this project could impact future practice, and indicated that podcasts may become crucial asynchronous learning tools in medical education.

**Results:**

Literature review and first-hand experience of podcast production enabled the students to appreciate the advantages of podcasting and the potential for its widespread future applications. Their wider reading revealed that podcast-using study participants outperformed or matched their peers in assessments, and overwhelmingly enjoyed using podcasts over traditional teaching methods.

**Conclusions:**

The use of podcasting can complement traditional psychiatry training and appeal to a generation of digital natives that prefer this learning style. Podcast production is also an excellent revision method, highlighting the advantages of peer-to-peer education in both learning and increasing engagement with psychiatry.

**Disclosure:**

No significant relationships.

